# The arrow of time of brain signals in cognition: Potential intriguing role of parts of the default mode network

**DOI:** 10.1162/netn_a_00300

**Published:** 2023-10-01

**Authors:** Gustavo Deco, Yonatan Sanz Perl, Laura de la Fuente, Jacobo D. Sitt, B. T. Thomas Yeo, Enzo Tagliazucchi, Morten L. Kringelbach

**Affiliations:** Center for Brain and Cognition, Computational Neuroscience Group, Department of Information and Communication Technologies, Universitat Pompeu Fabra, Barcelona, Spain; Institució Catalana de la Recerca i Estudis Avançats (ICREA), Barcelona, Spain; Department of Neuropsychology, Max Planck Institute for Human Cognitive and Brain Sciences, Leipzig, Germany; School of Psychological Sciences, Monash University, Melbourne, Clayton VIC, Australia; Department of Physics, University of Buenos Aires, Buenos Aires, Argentina; Sorbonne Université, Institut du Cerveau - Paris Brain Institute - ICM, Inserm, CNRS, APHP, Hôpital de la Pitié Salpêtrière, Paris, France; Centre for Sleep & Cognition, Centre for Translational MR Research, Department of Electrical and Computer Engineering, N.1. Institute for Health and Institute for Digital Medicine, National University of Singapore, Singapore; Latin American Brain Health Institute (BrainLat), Universidad Adolfo Ibanez, Santiago, Chile; Centre for Eudaimonia and Human Flourishing, Linacre College, University of Oxford, Oxford, UK; Department of Psychiatry, University of Oxford, Oxford, UK; Center for Music in the Brain, Department of Clinical Medicine, Aarhus University, Aarhus, Denmark

**Keywords:** Thermodynamics, Neuroimaging, Brain, Default mode network, Orchestration

## Abstract

A promising idea in human cognitive neuroscience is that the default mode network (DMN) is responsible for coordinating the recruitment and scheduling of networks for computing and solving task-specific cognitive problems. This is supported by evidence showing that the physical and functional distance of DMN regions is maximally removed from sensorimotor regions containing environment-driven neural activity directly linked to perception and action, which would allow the DMN to orchestrate complex cognition from the top of the hierarchy. However, discovering the functional hierarchy of brain dynamics requires finding the best way to measure interactions between brain regions. In contrast to previous methods measuring the hierarchical flow of information using, for example, transfer entropy, here we used a thermodynamics-inspired, deep learning based Temporal Evolution NETwork (TENET) framework to assess the asymmetry in the flow of events, ‘arrow of time’, in human brain signals. This provides an alternative way of quantifying hierarchy, given that the arrow of time measures the directionality of information flow that leads to a breaking of the balance of the underlying hierarchy. In turn, the arrow of time is a measure of nonreversibility and thus nonequilibrium in brain dynamics. When applied to large-scale Human Connectome Project (HCP) neuroimaging data from close to a thousand participants, the TENET framework suggests that the DMN plays a significant role in orchestrating the hierarchy, that is, levels of nonreversibility, which changes between the resting state and when performing seven different cognitive tasks. Furthermore, this quantification of the hierarchy of the resting state is significantly different in health compared to neuropsychiatric disorders. Overall, the present thermodynamics-based machine-learning framework provides vital new insights into the fundamental tenets of brain dynamics for orchestrating the interactions between cognition and brain in complex environments.

## Author Summary

Here we used a thermodynamics-inspired, deep learning based Temporal Evolution NETwork (TENET) framework to assess the asymmetry in the flow of events, ‘arrow of time’, in human brain signals. This was applied to large-scale HCP neuroimaging data which showed significant changes between the hierarchy of orchestration for the resting state and seven different cognitive tasks. Similarly, the hierarchy of the resting state is significantly different in health compared to neuropsychiatric disorders. This framework provided new insights into the orchestrating of brain dynamics in different brain states.

## INTRODUCTION

A major aim of cognitive neuroscience is to discover the physical underpinnings of cognition and behaviour. Early studies used recordings and lesions in animal models (LeDoux, 2000; Murray & Rudebeck, 2018), while human research has been mostly constrained to study the consequences of neurological disorders leading to relatively precise cognitive and emotional deficits (Adolphs, 2016). The invention of human neuroimaging started an avalanche of studies monitoring the changes in brain activity during cognitive tasks (Poldrack, 2012), which led to a better understanding of the processing in sensorimotor regions and during tasks. In addition, these studies curiously also led to the discovery of a network of regions deactivated during task (Raichle et al., 2001; Shulman et al., 1997), which came to be known as the default mode network (DMN) and which includes the precuneus/posteromedial cortex (PMC) and angular gyrus, regions of the inferior frontal gyrus, the medial prefrontal cortex (MPFC) and the anterolateral middle temporal cortex. Paradoxically, despite the DMN’s apparent deactivation during task, subsequent careful studies of the DMN have instead led to this network becoming a leading candidate for the orchestration of cognition during task (Raichle, 2006; Raichle et al., 2001). According to this view, the DMN is responsible for coordinating the recruitment and scheduling of networks computing and solving the task-specific cognitive problems (Smallwood et al., 2021). In other words, rather than the DMN being deactivated during tasks, findings from recent studies have suggested that in certain task contexts the DMN can *activate*, for example, during memory guided decision-making (Murphy et al., 2018, 2019). Note that this view is not incompatible with a *static* deactivation of the DMN during tasks over longer time periods (Raichle et al., 2001; Shulman et al., 1997) but could reflect a more dynamic view of how the DMN is stable across tasks and therefore able to orchestrate activity (Smallwood et al., 2021).

Importantly, Margulies and colleagues have demonstrated that the physical and functional distance of the regions of the DMN are maximally removed from sensorimotor regions containing extrinsically driven neural activity directly linked to perception and action (Margulies et al., 2016). This would make sense in terms of an evolutionary drive for more complex behaviour, more decoupled from the here-and-now and able to make long-term predictions assuring survival. As such, regions furthest away from externally driven regions would be able to take on roles that are both more complex and less directly influenced by the external environment, allowing for the orchestration of more complex behaviour (Buckner & Krienen, 2013).

Despite these important findings and hypotheses, we are still missing a better understanding and quantification of how the functional hierarchy changes when we engage in tasks compared to the resting state, both of which require cognition but of different kinds. Hierarchy can be defined from the causal interactions between different brain regions, which changes in tasks according to the required computations. This in turn changes the direction of information flow, which in effect means that the asymmetry in the underlying causal interactions change. Promising research has used a plethora of ways to directly quantify the hierarchy through determining the underlying causal interactions between brain regions, with methods ranging from Granger causality (Seth et al., 2013), transfer entropy (Brovelli et al., 2015; Deco et al., 2021b), and dynamic causal modelling (Frässle et al., 2017; Friston et al., 2003; Prando et al., 2020).

Here, we propose to use an alternative thermodynamics-inspired approach to determine the differences in hierarchical organisation in resting state and seven tasks. In other words, this method allows for the quantification of the hierarchy defined as the asymmetrical relationship between forward and backward interactions between brain regions.

The key idea is to be able to assess the asymmetry in the flow of events in human brain signals. In thermodynamics this is called ‘arrow of time’ and is a direct measure of hierarchy since this directly provides the *directionality of information flow*, or ‘breaking the detailed balance’ as this is known in physics and systems biology. In this way, a flat hierarchy is characterised by a low level of breaking the detailed balance, since the information flow is mostly symmetrical. When breaking the directionality of information flow, that is, when breaking the detailed balance, this results in a high level of hierarchical organisation.

Importantly, such processes happen at every level in biology, where all living systems must break detailed balance to survive. At a general level, the process of breaking the detailed balance is achieved by consuming energy and producing entropy through a whole host of molecular and cellular functions, including sensing, adaptation, and transportation (Lynn et al., 2021). It is important to realise the difference between *entropy* as a measure of disorder, that is, the variability of the states of a system, and the concept of production entropy, which directly measures the asymmetry in time of the evolution of the states in a nonequilibrium system. The latter is well suited to elucidate the differences in hierarchical organisation of different systems given that it quantifies the level of nonreversibility.

More specifically, our new framework estimates hierarchical organisation, not using the production entropy but using a direct way of measuring the ‘arrow of time’, central to thermodynamics in physics, which was originally popularised by Arthur Eddington (Eddington, 1928) and since studied in great detail in a number of fields (Crooks, 1998; Feng & Crooks, 2008; Jarzynski, 2011; Maragakis et al., 2008; Seif et al., 2021; Shirts et al., 2003). In the context of neuroscience, there has been considerable interest in using production entropy and related concepts to characterise the time reversibility of brain signals (Deco et al., 2022; Lynn et al., 2021; Palus, 1996; Sanz Perl et al., 2021; Zanin et al., 2019). However, there are significant statistical problems arising when the fluctuations are high, which makes it difficult to determine the direction of the arrow of time. Here, we applied the excellent idea of turning the quantification of the direction of arrow of time into a problem of statistical inference for a physical system (Crooks, 1998; Feng & Crooks, 2008; Jarzynski, 2011; Maragakis et al., 2008; Seif et al., 2021; Shirts et al., 2003). Among others, Seif and colleagues demonstrated that deep learning can be used to measure the arrow of time in forward and time-reversed time series, compare the two, and provide a quantitatively measure of the reversibility of signals (Seif et al., 2021). They were able to show that deep learning is able to capture time’s arrow in relatively simple physical systems where the ground truth is known.

Here we use a deep learning for human brain signals using a Temporal Evolution NETwork (TENET) framework to discover the asymmetry in the flow of events, that is, arrow of time. The face validity of this approach has been demonstrated in the pioneering work by Seif, Hafezi, and Jarzynski using two model physical examples in nonequilibrium (Seif et al., 2021). Please note that recent progress in thermodynamics has allowed for the study describing the dynamics of open systems driven out of equilibrium rather than merely isolated systems (Jarzynski, 2011; Seif et al., 2021).

Here, we wanted to identify the functional hierarchy of the brain at rest and during tasks. We achieved this by using the TENET framework to assess the level of nonreversibility (arrow of time) in brain dynamics during the resting state and seven different tasks from the large Human Connectome Project (HCP) neuroimaging fMRI dataset of 990 healthy human participants. TENET was trained on the data from 890 participants, and the results were generated from a generalisation set of data from the remaining 100 randomly selected participants. The same 100 participants were used for all comparisons across conditions.

Given the importance of hierarchical organisation for the successful orchestration of a given brain state, we wanted to discover how the functional hierarchy changes in neuropsychiatric disorder. We therefore used exactly the same approach to study time’s arrow in health and disease in a UCLA dataset of 261 neuropsychiatric patients (ADHD, schizophrenia, and bipolar disorder as well as controls). Here, again TENET was trained on 90% of the data, and the results were generated using a generalisation set of data from the remaining 10% of the data.

The results showed that in healthy participants, the whole-brain levels of nonreversibility are at higher levels during task than when resting. Interestingly, in neuropsychiatric disorders, the brain is less hierarchical with lower levels of nonreversibility during resting state than in healthy individuals, suggestive of less specific computation. In disease, brain processing is less efficient, which is reflected in a lower level of asymmetry of interactions between brain regions, resulting in a different hierarchical organisation leading to the lower levels of nonreversibility. In healthy participants, we also found significant differences at the system level and regional levels between resting state and the different tasks. Most importantly, consistent with other compelling anatomical and functional neuroimaging findings, we were able to demonstrate that across the seven tasks, DMN contains the most endogenous regions in terms of stability across conditions. This suggests that key DMN regions can be found at the top of the brain hierarchy, providing some support for the hypothesis that the DMN is involved in orchestrating cognition. Overall, beyond identifying the potential role of the DMN, TENET provides a general, convenient framework for assessing the functional hierarchy in any given brain state.

## RESULTS

Brain hierarchy can be defined as the asymmetrical relationship between forward and backward interactions between brain regions. TENET uses the thermodynamic concept of ‘arrow of time’ (also known as nonreversibility, or irreversibility) to reveal the hierarchical brain organisation in different brain states. In the language of thermodynamics, this estimates the ‘breaking of the detailed balance’ in the time series across the whole brain. The second law of thermodynamics states that production entropy increases over time, including in an open nonequilibrium system, such as the brain. The Clausius inequality of classical thermodynamics predicts that the external work performed on the system will be no less than the free energy difference between the terminal states (Jarzynski, 2011). This inequality precisely links production entropy and nonreversibility, establishing the arrow of time in nonequilibrium systems (Jarzynski, 2011; Seif et al., 2021). This has been extensively used for problems related to thermodynamics of system in nonequilibrium including biological problems such as protein folding (Collin et al., 2005). Indeed, the production entropy is a measure of nonreversibility and arrow of time, when defined as the Kullback–Leibler distance *H*_*P*_ = ∑_*i*,*j*_ *P*_*ij*_log(*P*_*ij*_/*P*_*ji*_), where *P*_*ij*_ is the probability of transition between states *i* at time *t* to *j* at time *t* + 1. In other words, production entropy is directly measuring the difference between forward and backward evolution of states over time.

Here, rather than directly estimating the production entropy, which as mentioned earlier is very difficult, we created a deep learning based Temporal Evolution NETwork (TENET) framework to discover the asymmetry in the flow of events, that is, ‘arrow of time’, in human brain signals. TENET was used in different brain states in health and disease to provide a quantification of the role of the DMN in orchestrating cognition.

**Figure 1.**
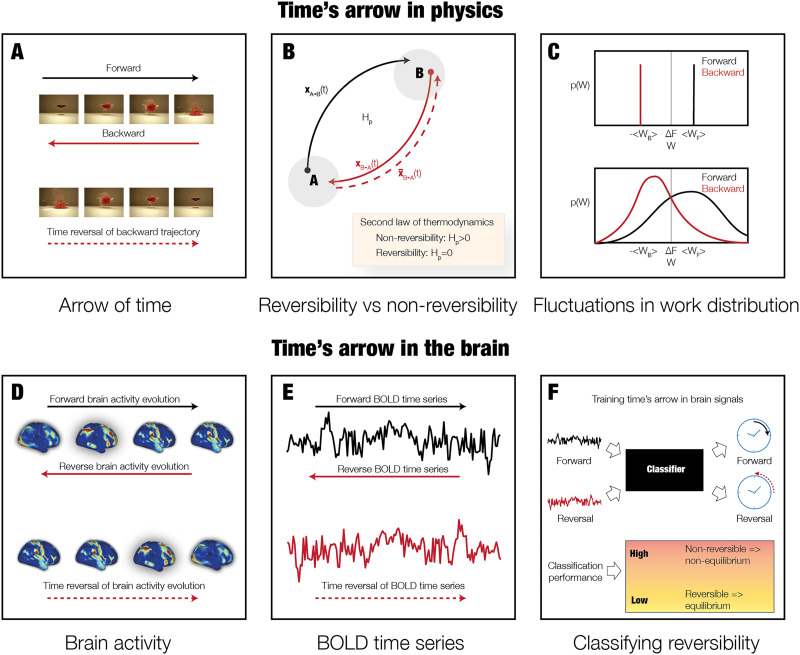
The arrow of time in physics and brain dynamics. (A) The sequence of the four top images shows a glass being shattered by a bullet, and we clearly perceive the causal passage of time, also called the arrow of time. In contrast, this cause and effect is shattered by showing these images backward—by time reversing the backward evolution. This means that this process is nonreversible. (B) In thermodynamics, nonreversibility can be associated with the production of entropy. The figure shows a nonequilibrium system with two states A and B and the associated trajectories evolving during forward (A → B, black arrow) and backward (B → A, red arrow) processes. Both the forward and backward trajectories can be depicted as the movie shown in the top of panel A, but with a different arrow of time. In contrast, the time reversal of the backward trajectory (red stippled arrow) can be imagined as the movie of the backward trajectory that is played forward in time (see bottom of panel A). If the forward and time reversal of the backward trajectories are different, this corresponds to nonreversibility of the process. The second law of thermodynamics uses the entropy production to describe this. If the entropy production is larger than zero, this corresponds to nonreversibility of a nonequilibrium system. In contrast, if there is no entropy production, this is a reversible, equilibrium system. (C) More specifically, when small systems undergo thermodynamic processes, the fluctuations are nonnegligible and the second law of thermodynamics expresses this in terms of averages. (D) First, we used large-scale empirical whole-brain neuroimaging data from over 1,000 participants when resting and performing seven different tasks. (E) From this data, we were able to extract the forward time series as well as constructing the time reversal of the backward time series for a given parcellation. (F) This procedure provides a clear arrow of time for a given time series and allows us to train a classifier to identify the forward and the time reversal for a given time series of any length. The classification performance provides a measure of the degree of nonreversibility and nonequilibrium.

**Figure 2.**
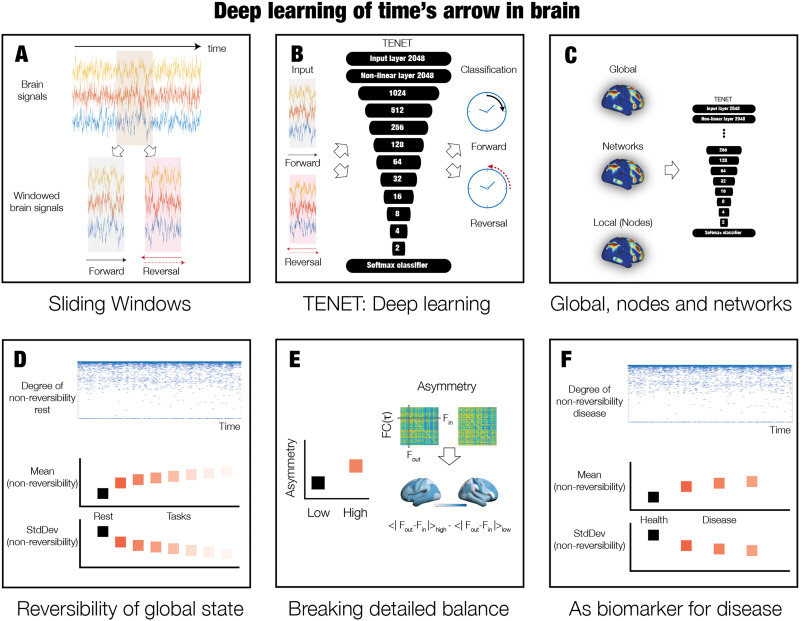
Deep learning the arrow of time in health and disease. In order to discover the arrow of time in brain dynamics in health and disease, we designed a deep-learning pipeline named Temporal Evolution NETwork (TENET). (A) Specifically, we used sliding windows of brain signal time series from all brain regions in all participants. (B) These sliding windows were then used in the TENET, a deep-learning network classifier with 13 layers for classifying the arrow of time. (C) This strategy allowed us to study nonreversibility and nonequilibrium at different levels of granularity, from global (all signals) to system level to individual node-level signals. (D) After training, TENET was able to characterise the degree of reversibility, that is, nonreversibility for each sliding window (top panel). We performed this procedure on data resting and seven tasks and computed the means of the levels of certainty of the classifier output (across time) as a measure of the degree of nonreversibility (middle panel). The standard deviation of this measure establishes stability of this nonreversibility across time. Given that nonequilibrium states are already nonstationary, this provides the second order of nonstationarity (see Methods). (E) Nonequilibrium is associated with the breaking of detailed balance of a system. We estimated this by selecting windows of low and high reversibility, and computing the FC(*τ*), that is, the time-delayed functional connectivity between all pairs of brain regions. Specifically, the degree of asymmetry of the FC(*τ*) matrix is a proxy for the breaking of the detailed balance with more asymmetry corresponding to more unbalance. The level of asymmetry can also be rendered on the brain (see Methods). (F) Finally, we used TENET on resting-state data from neuropsychiatric patients with diagnoses of schizophrenia, ADHD, and bipolar disorder, as well as age-matched controls. Different levels of nonreversibility provide a potential biomarker of neuropsychiatric disease.

Figure 1 and Figure 2 provide a schematised version of the general TENET paradigm used here. The key concept of the arrow of time in nonequilibrium systems is demonstrated in Figure 1A, which shows four sequential images from a film of a glass being shattered by a bullet. Below, the same four images are shown in a sequence in an opposite direction, that is, in time reversal of the backward trajectory of the film. When comparing the two films, the arrow of time is very clear, which is the signature of a nonreversible physical process producing entropy in nonequilibrium. More general, as shown in Figure 1B, the field of thermodynamics in physics can be used to describe such processes associated with the production of entropy and consequently with nonequilibrium/nonreversibility. The figure shows the evolution over time of a nonequilibrium system with two states A and B and their associated trajectories. The forward and backward trajectories of the movies in Figure 1A are described as forward (A → B, black arrow) and backward (B → A, red arrow) processes. The time reversal of the backward trajectory (red stippled arrow) can be thought of as the movie of the backward trajectory that is played forward in time (see bottom of Figure 1A). A nonreversible process results from the ability to differentiate between the trajectories in time described by the forward (black arrow) and time reversal (stippled red arrow). The second law of thermodynamics (usually attributed to Rudolph Clausius and Sadi Carnot) states that if the entropy production is larger than zero, this corresponds to nonreversibility of a nonequilibrium system. In contrast, if there is no entropy production, this describes a reversible, equilibrium system.

In thermodynamics, the Clausius inequality establishes that the work W associated with the process (averaged over many repetitions) is larger than the change in its free energy ΔF. Figure 1C shows distributions of the work p(W) for the average of the work associated with the forward and backward trajectories, denoted <W_F_> and <W_B_>, respectively. For nonreversible macroscopic processes (like the movie shown in Figure 1A) fluctuations are negligible and the distinction is clear between the distribution of work (top of panel) and therefore the arrow of time is easy to establish. In contrast, in microscopic systems (which includes brain signals) the average work is similar, but the fluctuations are more pronounced and therefore the differences in distribution less clear. In such cases it is much harder to establish the arrow of time, and thus establish whether a system is nonequilibrium and nonreversible.

This uncertainty is a perfect case for which to use advanced machine-learning techniques (Sejnowski, 2018). Here, we used deep learning in empirical brain-imaging data to detect the reversibility of the system. Figure 1D and Figure 1E illustrate how we used whole-brain activity from large-scale empirical whole-brain neuroimaging data from over 1,000 participants to construct the forward and time-reversed time series needed to establish the arrow of time and hence nonequilibrium by detecting the level of nonreversibility. Specifically, Figure 1F illustrates how we extracted the forward time series as well as constructing the time reversal of the backward time series for the DK80 parcellation (see Methods). The forward and reverse time series were used to train a classifier to predict whether a given time series is forward or reversed in time. If the classification performance is high, this provides evidence for nonreversibility and nonequilibrium, while low performance implies the opposite. We hypothesized that brain regions at the bottom of the functional hierarchy will exhibit greater nonreversibility given that these regions will be driven to nonequilibrium. Therefore, this approach can be used to identify the functional hierarchy of the human brain.

Figure 2 specifies the full learning pipeline using a deep-learning TENET to establish the arrow of time. Figure 2A shows how we used sliding windows of brain signal time series from all brain regions in all participants. Figure 2B shows how these sliding windows were then used in TENET, a deep-learning network classifier with 13 layers for classifying the arrow of time. Figure 2C shows how this strategy allowed us to study nonreversibility and nonequilibrium at different levels of granularity, from global (all signals) to system level to individual nodes. TENET allows to quantify the information transfer *within* the levels of granularity but not *between* levels.

Figure 2D shows how TENET should be able to characterise the degree of reversibility, that is, nonequilibrium for each sliding window (top panel). We trained the TENET on a large dataset of data resting and seven tasks and, on a validation dataset, computed the means of the prediction performance of the classifier output (across time) as a measure of the degree of nonreversibility (middle panel). Importantly, all datasets were shortened to the same task duration to avoid the potential confound of one condition gaining undue prominence due to more data being available. The standard deviation of prediction performance establishes stability of this nonreversibility across time. Given that nonequilibrium states are already nonstationary, this provides the second order of nonstationarity (see Methods).

Figure 2E shows that nonreversibility is associated with the breaking of detailed balance of a system. In order to test the breaking of the detailed balance of the system, we selected windows of low and high reversibility and computing the time-delayed functional connectivity between all pairs of brain regions. Specifically, the degree of asymmetry of this matrix is a proxy for the breaking of the detailed balance with more asymmetry corresponding to more unbalance. Finally, Figure 2F shows how TENET can be used on resting-state data from neuropsychiatric patients with diagnoses of schizophrenia, ADHD, and bipolar disorder, as well as age-matched controls. Importantly, computing the different levels of nonreversibility could provide a potential biomarker of neuropsychiatric disease and reveal the underlying fundamental problem with interacting with the environment.

In the following, we established the role of the DMN in cognition by applying the TENET framework in healthy participants engaged in rest and seven tasks. We first show the results at the whole-brain level, followed by the system level and the regional node level. Finally, we apply the TENET framework to resting state in three neuropsychiatric disorders.

### Significant Global Differences in Brain-Environment Interactions for Rest and Seven Tasks

For the global level of analysis of how the environment is driving the brain out of equilibrium, we extracted BOLD time series from the DK80 parcellation covering the whole brain in rest and the seven tasks. For each of HCP participant, we extracted forward and backward patterns in sliding windows with a length of 20 TRs (of 0.72 sec), which were then shifted 3 TRs forward. Each of the sliding windows consisted of two input patterns containing (1) forward and (2) time-reversed backward-sliding windowed time series, which was each labelled with an output class label of forward and backward, respectively.

For the training of TENET, in order to perfectly balance the data and avoid any potential source of bias, we used 890 HCP participants with the longest possible duration available in all conditions (176 TRs). For generalisation, we performed the data analysis on a separate 100 HCP participants. The data analysis consisted of computing the level of nonequilibrium/nonreversibility, *R*(*t*), using the output of TENET on this generalisation set after being trained on the bulk of the data.

As specified in detail in the Methods, *R*(*t*) is computed as the accuracy of classification of forward and time reversal of backward trajectory of the global time series (across sliding windows at time *t* and across participants). Perfect classification of maximal nonreversibility is thus assigned a value of 1 and where 0 corresponds to full reversibility.

**Figure 3.**
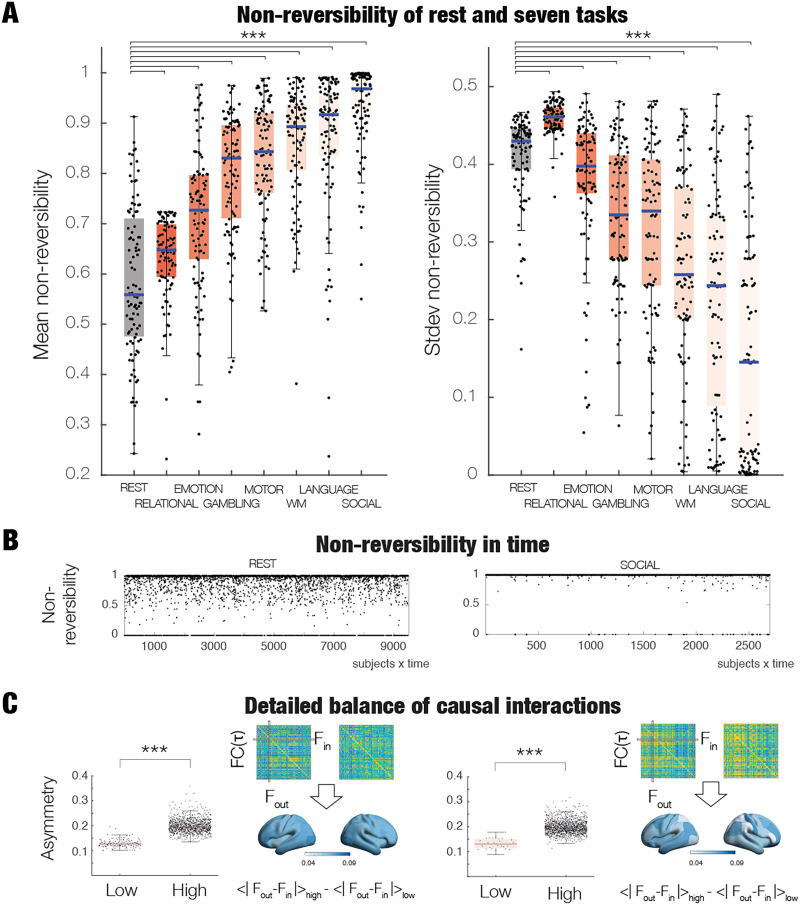
Global nonreversibility in HCP rest and seven tasks. (A) Left panel shows the mean nonreversibility for rest and the seven tasks ordered by the increase in their mean level of nonreversibility. The level of nonreversibility, *R*(*t*), is computed using the output of TENET on a 10% validation set after being trained on 90% of the data. In brief, *R*(*t*) is computed as the accuracy of classification of forward and time reversal of backward trajectory of global timeseries (across sliding windows at time *t*), where a value of 1 corresponds to perfect classification, that is, maximal nonreversibility (see Methods). As can be seen from the box plot, brain dynamics during rest exhibits significantly lower levels of reversibility than that found in tasks (all *p* < 0.01, Wilcoxon rank sum, corrected for multiple comparisons with FDR). The highest level of nonreversibility is found in the Social task, reflecting a stronger arrow of time. In other words, the brain dynamics in tasks are showing more nonreversibility than rest and therefore more hierarchical organisation underlying specific computations. The right panel shows the stability of this nonreversibility across time, that is, providing a measure of second order of nonstationarity. Brain activity during rest is showing significantly more variability in the second order of nonstationarity than tasks (all *p* < 0.01, Wilcoxon rank sum, corrected for multiple comparisons with FDR). (B) The panel shows the level of nonreversibility, *R*(*t*), over time for rest (left panel) and the social task (right panel). Note how the evolution of *R*(*t*) is more variable in rest. (C) Interactions vary across time and consistently show a significantly stronger breaking of the detailed balance in windows with high compared to low levels of nonreversibility (compare low and high box plots, *p* < 0.001) for both rest (left panel) and the social task (right panel). This is measured as the asymmetry of the time-shifted functional connectivity (see Methods). The renderings of brains reflect which brain regions are showing more symmetry breaking between low and high levels of nonreversibility. The brain shows more heterogenous patterns of change during the social task than in rest (compare right with left panel).

Figure 3A (left panel) contains a box plot showing that the brain dynamics during REST have significantly lower levels of reversibility than in tasks (all *p* < 0.01, Wilcoxon rank sum, corrected for multiple comparisons with FDR). As can be seen, the highest level of nonreversibility is found in the SOCIAL task, reflecting how the environment is forcing a stronger arrow of time and thus nonreversibility. But, equally, the other tasks, ordered by levels of nonreversibility (RELATIONAL, EMOTION, GAMBLING, MOTOR, WM (working memory) and LANGUAGE) are significantly more hierarchically structured than REST, related to the increase in the breaking of the balance directly related to the necessity of more structured computation. It is interesting to note the significant differences between the tasks too (all *p* < 0.01, Wilcoxon rank sum, corrected for multiple comparisons with FDR; all significant comparisons between conditions except for MOTOR vs. LANGUAGE, MOTOR vs. WM, and WM and GAMBLING).

In addition, the right panel shows the level of nonstationarity, which is the standard deviation of the levels of nonreversibility across time. The differences between rest and tasks were similar to the results of the mean of the nonreversibility in the sense that there were significant differences between all conditions (all *p* < 0.01, Wilcoxon rank sum; corrected for multiple comparisons with FDR, except between MOTOR vs. LANGUAGE and WM and GAMBLING), but importantly for this measure, the SOCIAL task had the lowest variability over time, which was much lower than REST. On the other hand, REST is showing one of the largest levels of nonstationarity, which is consistent with the idea that resting state involves less computation and thus less breaking of the detailed balance. This can also be appreciated from Figure 3B, where the two panels show time evolution of the levels of nonequilibrium/nonreversibility, R(t), for REST (left) and the SOCIAL task (right).

Please note that the increase in nonreversibility during tasks, linked to the increase in production entropy, is a measure of the increase in asymmetrical interactions. This should not be confounded with the findings of a *decrease* in entropy for tasks found in the literature (He, 2013; Ponce-Alvarez et al., 2015). The entropy measured in these studies is a measure of the variability of the state, which has been shown to decrease in task. As such, entropy and production entropy are complementary measures of the system.

### Differences in Asymmetry Breaking Between Rest and Tasks

As mentioned above, equilibrium is associated with the fluxes of transitions between different states, that is, how the detailed balance of the transitions between the underlying states disappear in completely equilibrium. In thermodynamics, a nonequilibrium system contains net fluxes between the states as a function of broken balance, which is the source of nonreversibility and thus of the arrow of time (Crooks, 1998; Feng & Crooks, 2008; Maragakis et al., 2008; Seif et al., 2021; Shirts et al., 2003). In order to establish a quantitative link between our measure of nonequilibrium/nonreversibility and broken detailed balance, we measured the asymmetry of the time-shifted functional connectivity (see Methods).

In brief, in order to measure a proxy for the causal interactions, we selected patterns from sliding windows of low and high reversibility, and computed the time-delayed functional connectivity matrix, *FC*(*τ*), between all pairs of brain regions, over all participants and all sliding windows for each condition of HCP REST and the SOCIAL task, which is the task with the most nonreversibility. The global level of asymmetry was computed for each sliding window as the mean value over the elements of the difference between this matrix and its transposed. In contrast, for the node level of asymmetry, we first computed the incoming and outgoing regional flow for each sliding window and then computed the average over all sliding windows and participants of the absolute difference between the two regional flows (see Methods for detailed information). We render the change between high and low levels of the node-level asymmetry.

As can be seen in Figure 3C, we found significantly stronger breaking of the detailed balance in windows with high compared to low levels of nonequilibrium/nonreversibility (compare low and high box plots, *p* < 0.001, Wilcoxon rank sum). On the right of the box plot, we show an example of the asymmetry matrices for a single participant at a given time point. Below renderings are shown of the change between low and high levels of the node-level asymmetry.

Consistent with the close link between symmetry breaking and our measure, we found more heterogenous patterns of change during the SOCIAL task than in REST. This again demonstrates that when engaged in a task, the environment is driving the brain in very specific ways to higher levels of nonreversibility. Please note, however, that in general, the breaking of the detailed balance reflects the level of nonreversibility, although there could, of course, be rare cases of systems where the absence of directed information flow could still lead to nonreversibility (Ao, 2008).

Please note that the findings of TENET that tasks have higher nonreversibility than resting state make it highly unlikely the results are biased by the onset of task blocks. Four lines of argumentation supports this: (1) The asymmetry of the HRF associated with task blocks could be a potential confound, especially if the windows used for classification are synchronised with the task blocks. However, crucially, here we are using the same windows for the classification of rest and all tasks, where the task onsets are completely different. Furthermore, similar to tasks, resting state is also composed of spontaneous neuronal activity, which is also convolved with HRF. Ultimately, if HRF was a confound, it would not be specific to task or rest. In fact, there is a large literature showing how resting state can be obtained using neural discrete event convolution (e.g., Tagliazucchi et al., 2016). (2) The results show that the SOCIAL task is the task with the highest nonreversibility and RELATIONAL has the lowest. Yet, SOCIAL has less blocks than RELATIONAL, making it unlikely that task blocks are important for the estimation of reversibility. (3) TENET uses sliding windows of 20 TRs (14 sec) with increase of 2 or 3 TRs, which are smaller than most task blocks and makes it unlikely that the results are biased by the task blocks. (4) As shown in Figure 3C, the windows with high nonreversibility are the ones with maximal asymmetry. Note that this shows that the temporal asymmetry correlates with the hierarchical organisation, that is, asymmetry of interactions. This is consistent with the examples using spin models used by Lynn and colleagues, showing that nonreversibility (production entropy) correlates with the breaking of the detailed balance, that is, the asymmetry of interactions. In other words, different tasks require different hierarchical organisation (asymmetry interactions), which is detected by the nonreversibility.

### System-Level Analysis of HCP Data Shows That DMN is the Most Endogenous Network

In order to assess the system level of the brain-environment interactions between rest and task, we used TENET framework in the same manner as in the global analysis, but now used on the parcels belonging to each Yeo network in the Schaefer 500 parcellation. Again, in order to balance the data, we used 890 HCP participants with the longest possible duration available in all conditions (176 TRs). The results are from the generalisation that was performed on a separate 100 HCP participants (see Methods). We used the same sliding window size and shifting of this window as in the global-level analysis, but now the input is the window size multiplied by the number of parcels for a given level analysis of HCP rest and tasks. Analysis of the nonreversibility of the seven Yeo resting-state networks showed differential responses between rest and tasks for the seven resting-state networks.

**Figure 4.**
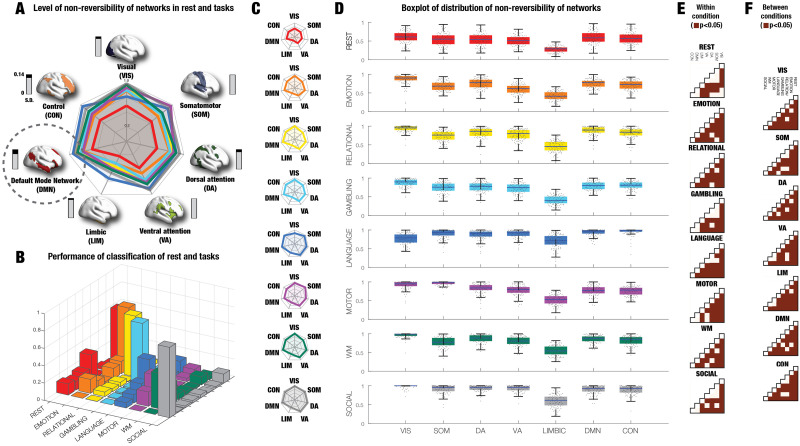
TENET system-level analysis of HCP rest and tasks. Similar to the whole-brain level analysis, we found different brain-environment interactions between rest and task at the system level. Here our focus was on revealing how the seven Yeo networks are changing between rest and tasks. (A) The combined spider plot shows the different levels of nonreversibility for each Yeo network in rest and the seven tasks. Each Yeo network is rendered on the brain with a separate colour coding. Most importantly, the bar next to the rendering shows the standard deviation across rest and tasks (ranging from 0 and 0.14). Importantly, the smallest standard deviation is found for DMN (circled), which is therefore the most endogenous network. This can be linked with the compelling anatomical and functional neuroimaging evidence from Margulies and others showing that the physical and functional distance of the regions of the DMN are maximally removed from sensorimotor regions (containing extrinsically driven neural activity directly linked to perception and action). Together this provides evidence that the DMN is a strong candidate for orchestrating cognition. (B) The system-level TENET results also allowed for a classification of conditions (rest and seven tasks). As can be seen from the confusion matrix, the SVM provides excellent classification results much above chance level. Average classification for the diagonal is 59% with a chance level of 12.5%. (C) Further probing the nonreversibility of the seven Yeo resting-state networks is demonstrated by differential responses between rest and tasks for the seven resting-state networks shown by the spider plots of the level of nonreversibility for each Yeo network in rest and seven tasks (colour coded similar to panel A). (D) This can be seen even more clearly in the box plots (for the validation data), where, similar to the global-level analysis, there are lower levels of nonreversibility for rest compared to the seven tasks, suggesting, as expected, that REST is more intrinsic and thus more in equilibrium. It is of interest to note that REST is characterised by having the highest levels of nonreversibility in the DMN and Visual (VIS) network. Equally, across the tasks, except for LANGUAGE, the sensory networks (VIS and SOM) show the highest level of nonreversibility. Interestingly, again except for LANGUAGE, the limbic network (LIM) exhibit the lowest levels of nonreversibility. Overall, of the seven tasks, the Yeo networks in EMOTION and RELATIONAL show almost as low levels of nonreversibility levels as REST. (E) In order to show the statistical significance, we show the comparisons between the level of nonreversibility of the seven Yeo networks within condition (rest and the seven tasks). The figure shows the significance in the lower quadrangle of the matrices with brown squares signifying *p* < 0.05, Wilcoxon rank sum. As can be seen almost all comparisons within conditions are significant but less so for REST. (F) Similar, we show the statistical significance between conditions for the level of nonreversibility of the seven Yeo networks. The figure shows the lower quadrangle of the matrices (with brown squares signifying *p* < 0.05, Wilcoxon rank sum). Almost all comparisons across conditions are significant.

Figure 4A shows the combined spider plot of the different levels of nonreversibility for each Yeo network in rest and the seven tasks, with a rendering of each Yeo network using separate colour coding. The bar next to the renderings shows the standard deviation across rest and tasks (ranging from 0 and 0.14). Importantly, the smallest standard deviation is found for DMN (circled), which is therefore the most stable and endogenous network. This corroborates the compelling anatomical and functional neuroimaging evidence from Margulies and others showing that the physical and functional distance of the regions of the DMN are maximally removed from sensorimotor regions (containing extrinsically driven neural activity directly linked to perception and action). Taken together this points to DMN being a strong candidate for orchestrating cognition.

Further, we investigated the possibility for classifying the conditions based on the system-level TENET output. Using a support vector machine (SVM) with Gaussian kernels on the 100 HCP participants used for generalisation. For the SVM, we subdivided the 100 participants into 90% training and 10% validation, repeated and shuffled 100 times. The SVM had seven inputs (the Yeo resting-state networks) corresponding to the output produced by the system-level TENET. The output was eight classes corresponding to the conditions (rest and seven tasks). Figure 4B shows the resulting confusion matrix, which provides excellent classification results much above chance level with an average classification accuracy of 59% (on the diagonal) compared with the chance level of 12.5% (using permutation testing; see Methods). Interestingly, the results of classifying rest versus all tasks, produced a very high accuracy of 93.1% on the generalisation dataset, using exactly the same procedure as for classifying the individual tasks.

Figure 4C and 4D show the differential responses between rest and tasks for the seven resting-state networks by presenting the spider plots and box plots of the level of nonreversibility for each Yeo network in rest and seven tasks (for the validation data, colour coded similar to Figure 4A). Similar to the global-level analysis, the lower levels of nonreversibility were found for rest compared to the seven tasks, suggesting that REST is more reversible and therefore less hierarchical. Overall, of the seven tasks, the Yeo networks in the tasks EMOTION and RELATIONAL show almost as low levels of nonreversibility levels as REST. Interestingly, the VIS and SOM networks exhibit very high levels of nonreversibility in all tasks, mostly likely reflecting the computational requirements for the sensory regions to work out the complexity of the environment. Similarly, the LIMBIC network has lower levels of nonreversibility in all tasks, perhaps reflecting the intrinsic nature of this network.

Figures 4E and 4F show the statistical significance within and across conditions, respectively. Both figures show the significance in the lower quadrangle of the matrices with brown squares signifying *p* < 0.05, Wilcoxon rank sum. As can be seen almost all comparisons within and across conditions are significant.

### Node-Level Analysis of Healthy Individuals Reveals DMN Orchestration of Cognition

Beyond the global and system-level analyses, we were interested in studying what endogenous brain regions are common across rest and tasks, and thus able to orchestrate cognition independently of the environment.

To this end, we applied the TENET framework at the node level using exactly the same amount of data across rest and seven tasks, similar as above (see Methods). For the training of TENET, in order to perfectly balance the data and avoid any potential source of bias, we used 890 HCP participants with the longest possible duration available in all conditions (176 TRs). For generalisation, we performed the data analysis on a separate 100 HCP participants.

**Figure 5.**
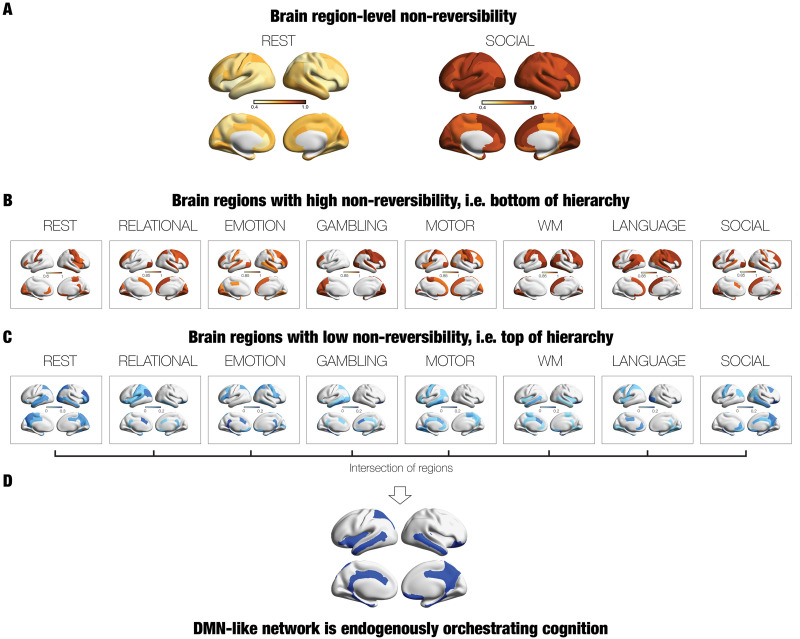
Node-level analysis reveals that a DMN-like network is endogenously orchestrating cognition. Applying TENET framework at the brain region level can distinguish the hierarchical organisation between rest and tasks. (A) The figure shows a brain rendering of the mean node-level nonreversibility (across participants) for resting and the social task, which show the lowest and highest levels of global nonreversibility, respectively. This is equally true at the node level but with significant interregional heterogeneity (compare the different shades in a common colour map from yellow to brownish red). (B) The figure shows the upper 30% quantile levels of nonreversibility for brain regions in rest and all the seven tasks, which shows that sensorimotor regions clearly at the bottom of the hierarchy. There are also clear differences between the tasks. For instance, the working memory (WM) task shows high levels of nonreversibility in prefrontal regions, while the LANGUAGE task shows high levels of nonreversibility in known language areas, consistent with the existing extensive literature. (C) In contrast to the previous renderings, this figure shows the brain regions at the top of the hierarchy, that is, more endogenous, rendering the lower 30% quantile levels of nonreversibility for rest and all the seven tasks. In the rendering, darker blue colours are more endogenous and thus at the top of the hierarchy. (D) Combining these eight conditions (rest and seven tasks) by selecting the intersection (see text) revealed that a set of regions (including precuneus/posteromedial, temporal, and ventromedial orbitofrontal cortices), mostly overlapping with the DMN is common across all conditions and thus orchestrating cognition.

The node-level rendering for nonreversibility for REST and the SOCIAL task is shown in Figure 5A. Similar to the global results, these are the two conditions with the lowest and highest levels of nonreversibility (compare lighter shades of brown for REST to the darker for SOCIAL). However, here we were also able to draw out the interregional heterogeneity. To further draw out the differences between tasks, in Figure 5B we render the thresholded node level of nonreversibility for all the seven tasks (thresholded to include the upper 30% quantile). This demonstrates that sensorimotor regions are among the most stable regions and that there are clear differences between the tasks.

Our main goal here, however, was to identify the endogenous regions at the top of the hierarchy. Figure 5C shows the lower 30% quantile levels of nonreversibility for rest and all the seven tasks, where darker blue colours show the less nonreversible brain regions. Note how these regions are primarily located in higher order regions on the midline of the brain.

Confirming this finding, Figure 5D shows the brain regions common to the eight conditions (rest and seven tasks) by selecting the lower 25% quantile levels for each condition and computing the intersection between conditions for brain regions with low nonreversibility, that is, top of hierarchy. This revealed a set of brain regions (including precuneus/posteromedial, temporal, and ventromedial orbitofrontal cortices, mostly overlapping with the DMN), which are common across all conditions and thus orchestrating cognition independently of the environment. Interestingly, this intersection also included regions not normally associated with the DMN such as the insula and superior parietal cortex.

In Figure S1 we further investigated these finding, demonstrating that our new measure characterises the engagement across the whole brain rather than just in sensory regions. We compared them directly to the myelinisation ratio (T1w/T2w ratio, obtained from HCP data), which contains high values in the sensory regions of visual, somatomotor and auditory (Burt et al., 2018). The nonsignificant correlations between the node levels of nonreversibility for both REST and SOCIAL task (left row) with this map could indicate that the new measure is not just linked to sensory but primarily to higher associative brain regions across the whole brain. On the other hand, computing the full intersection as the mean across all conditions and correlating this with the myelinisation ratio yielded a significant correlation (*r* = 0.26, *p* < 0.05, nonparametric). Given that the top of the hierarchy of the intersection (Figure 5C) consists of key regions in the DMN, the correlation with myelinisation provides further evidence for the role of the DMN in orchestrating brain function.

Further investigating links to other source of heterogeneity led us to investigate the various forms of gene expressions in the brain as obtained from the Allen Human Brain Atlas (Arnatkeviciute et al., 2019; Deco et al., 2021a; Fornito et al., 2019; Hawrylycz et al., 2012). The middle row of Figure S1 shows the correlations between and the first PCA component of all genes and the node level of nonreversibility of REST (top) and SOCIAL task (bottom). Interestingly, there was a significant correlation between the PCA genes values and node levels in the SOCIAL task (*r* = 0.47, *p* < 0.001, nonparametric), but not with the node levels in REST. Again, there was a significant correlation (*r* = 0.48, *p* < 0.001, nonparametric) with the intersection between conditions for brain regions with low nonreversibility, that is, top of hierarchy.

We also investigated another major source of heterogeneity, namely, the excitation-inhibition (E-I) ratio given by the gene expression for genes coding for the excitatory AMPA and NMDA receptors and inhibitory GABA-A receptor isoforms and subunits. In contrast to the PCA maps, the rightmost row of Figure S1 shows a significant correlation between the node level of nonreversibility in REST and the E-I values (*r* = 0.23, *p* < 0.04, nonparametric) but not for the node levels in the SOCIAL task.

### Using the Arrow of Time in Neuropsychiatric Disease

Given that the TENET framework by design measures how the environment is driving the brain, and its high level of sensitivity demonstrated above, it would appear a promising avenue for better characterising the differences between health and neuropsychiatric diseases. We therefore applied the TENET framework on the large public UCLA dataset of neuropsychiatric patients with schizophrenia, bipolar, and ADHD and matched control group of participants.

**Figure 6.**
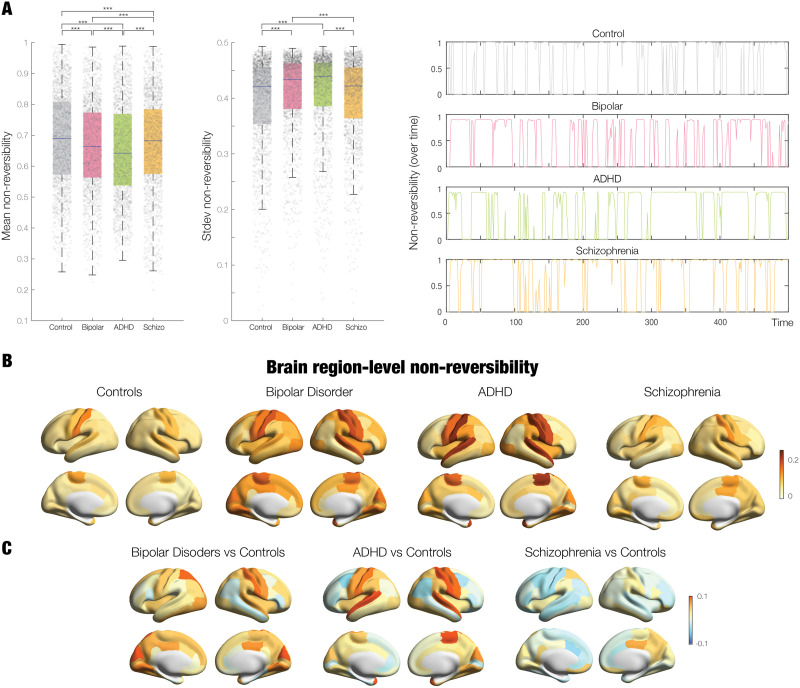
The arrow of time in neuropsychiatric disease. We used the nonreversibility on the global and local node levels on the large public UCLA dataset of neuropsychiatric patients with schizophrenia, bipolar, and ADHD and matched control group of participants. (A) First, we used the TENET framework to compute the level of nonreversibility at the global level for each group. The left panel of box plots shows that the average reversibility across time for the control group is significantly higher than each of the neuropsychiatric groups (all *p* < 0.05, Wilcoxon rank sum, corrected for multiple comparisons with FDR). In addition, each neuropsychiatric disease group are significantly different from each other (all *p* < 0.05, Wilcoxon rank sum, corrected for multiple comparisons with FDR). The middle panel of box plots shows that the standard deviation of the reversibility across time is significantly reduced for the control participants compared to neuropsychiatric groups and that between them, there are also significant differences (all *p* < 0.05, Wilcoxon rank sum, corrected for multiple comparisons with FDR, except for comparisons between controls vs. schizophrenia and bipolar vs. ADHD). The right panel shows examples of the temporal evolution of the reversibility computed by TENET for a participant from each of the four groups. (B) Complementing these findings at the global level, we used the TENET framework to compute the node-level reversibility for each group and show the corresponding thresholded renderings. (C) In order to stress the differences between the control group and the three neuropsychiatric disorders, we show renderings of these differences.

Figure 6 shows the results of using the TENET framework to establish the reversibility on the global and local node levels for the four groups. The left panel of Figure 6A shows box plots of the average reversibility across time, where the control group was significantly higher than each of the neuropsychiatric groups (all *p* < 0.05, Wilcoxon rank sum, corrected for multiple comparisons with FDR). This suggests that neuropsychiatric disease reduces the levels of nonreversibility, suggesting that the brain is less hierarchical. Furthermore, each neuropsychiatric disease group was significantly different from each other (all *p* < 0.05, Wilcoxon rank sum, corrected for multiple comparisons with FDR). Given that the median values are similar but significant, we also provide the effect size for each comparison: control versus bipolar: 0.1005; control versus ADHD: 0.1606; control versus schizophrenia: 0.0529; bipolar versus ADHD: 0.0693; bipolar versus schizophrenia: 0.0512; and ADHD versus schizophrenia: 0.1130.

The middle panel of Figure 6A shows box plots of the standard deviation of the reversibility across time for each of the four group, reflecting the levels of nonstationarity. This is significantly reduced for the control participants compared to neuropsychiatric groups and between them (all *p* < 0.05, Wilcoxon rank sum, corrected for multiple comparisons with FDR except controls vs. schizophrenia and bipolar vs. ADHD), that is, the brains of patients with ADHD and bipolar disorder are more nonstationary than controls. To further appreciate the differences between groups, right panel of Figure 6A plots examples of the temporal evolution of the global reversibility computed by TENET for all the four groups.

These promising results prompted us to use the TENET framework to compute the mean node-level reversibility for each group. Figure 6B shows the corresponding renderings on the human brain. We also computed the differences between the mean in the control group with the three neuropsychiatric groups, shown rendered in Figure 6C. As can be seen, and interpreted in details in the discussion, there are clear differences between groups, which suggest that the node level of nonreversibility might be useful as a biomarker for disease.

## DISCUSSION

Here we developed a thermodynamics-inspired, deep-learning TENET framework designed to identify the hierarchical organisation of any brain state. This allowed us to address a central, challenging problem in human cognitive neuroscience, namely, what brain networks are coordinating the recruitment and scheduling of networks for computing and solving task-specific cognitive problems. The leading hypothesis in human cognitive neuroscience is that the DMN is responsible for this orchestration (Smallwood et al., 2021). Using the TENET framework, we were able to demonstrate the breaking of detailed balance in rest and cognition. We found that an endogenous network largely overlapping with the DMN is responsible for the orchestration of cognition across rest and tasks. The TENET framework quantified the asymmetry in the flow of events, ‘arrow of time’, in human brain signals. The results reveal the independent, endogenous regions able to orchestrate activity, in contrast to the sensorimotor regions at the bottom of the hierarchy. These findings are in agreement with the findings of Margulies and colleagues, who demonstrated that the physical and functional distance of the regions of the DMN are maximally removed from sensorimotor regions (Margulies et al., 2016; Smallwood et al., 2021).

### Whole-Brain Differences in Brain-Environment Interactions in Health and Disease

We found higher global levels of nonreversibility in different tasks than during resting state in the large-scale HCP neuroimaging dataset (Figure 3). Similarly, we showed lower and less variable global levels of nonreversibility across time in three different neuropsychiatric patient groups (ADHD, schizophrenia, and bipolar disorder) in the large-scale UCLA neuroimaging dataset compared to healthy participants (Figure 6). This suggests a flattening of the functional hierarchy in disease, perhaps reflecting the lower asymmetry in functional interactions between brain regions. As speculation, this could directly influence the computation and lead to the more rigid and less flexible behavioural repertoire found in neuropsychiatric disorder.

### At the System Level, DMN is the Most Endogenous Network

Complementary to investigating the hierarchical organisation of brain states by quantifying the nonreversibility at the global level, we aimed to discover if there is more information to be extracted from the system level, specifically regarding differences between rest and task. We used the TENET framework on the seven Yeo resting-state networks in the Schaefer 500 parcellation. The results again showed lower levels of nonreversibility for rest compared to the seven tasks, suggesting that REST is more in equilibrium, while the tasks have higher levels of nonreversibility in the sensory networks (VIS and SOM).

Interestingly, the REST condition shows the highest levels of nonreversibility in the DMN and visual (VIS) network. Similarly, the sensory networks (VIS and SOM) are showing the highest level of nonreversibility across the seven tasks (except for LANGUAGE), and thus at the bottom of the hierarchy. The lowest levels of nonreversibility are found in the limbic network (LIM), signalling that the LIM network is less endogenous. Of the seven tasks the lowest levels of nonreversibility are found in the EMOTION and RELATIONAL tasks, which are almost as low as REST.

We wanted to measure the stability of the different networks across the conditions, which is captured by the standard deviation in the level of nonreversibility across time. In other words, having the smallest standard deviation across time implies stability. Importantly, as shown in Figure 4A in the combined spider plot in Yeo networks across all conditions, the DMN showed the smallest standard deviation. This confirmed that the DMN is indeed the most endogenous and at the top of the hierarchy, fitting well with the topographical evidence showing that the DMN is located in regions furthest away from those contributing to sensory and motor systems (Margulies et al., 2016; Smallwood et al., 2021). As such this provides further evidence that the DMN is strong candidate for leading the orchestration of cognition.

In addition, the system-level analysis can also be used to accurately classify condition. Using a SVM with Gaussian kernels to classify the conditions (rest and seven tasks), the results showed a very high accuracy of 59% compared to the 12.5% chance level. Equally, just classifying rest compared to all tasks the level of accuracy to 93.1%, showing that system-level TENET is an excellent method for distinguishing different cognitive brain states.

### Node-Level Identification of the Most Endogenous Brain Regions

We also used the TENET framework at the node level, which revealed that a network of endogenous brain regions largely overlapping with the DMN is orchestrating behaviour across rest and tasks. This provides further confirmation of the DMN as a strong candidate for leading the orchestration of cognition.

In contrast to these results investigating the more endogenous brain regions, we were also able to find brain regions with higher nonreversibility. Similar to the findings at the global scale, the findings at the local scale reveal clear interregional heterogeneities between rest and tasks (see Figure 5). The findings show that in many cases higher associative brain regions are more nonreversible during task performance in the HCP dataset. Specifically, Figure 5A shows the node-level rendering for nonreversibility for REST and the SOCIAL task. Like the results for the global level, these are the two conditions with the lowest and highest levels of nonreversibility.

The power of the TENET framework is perhaps illustrated in the results for the MOTOR task, where the thresholded results show selective engagement of the somatomotor regions as expected (Barch et al., 2013), but equally the results show engagement in visual cortices and midline medial prefrontal regions. Another good example is the LANGUAGE task, where the results show broad engagement of ventral lateral prefrontal cortex, superior and inferior temporal cortex—including the anterior temporal poles bilaterally (Barch et al., 2013; Binder et al., 2011). Also, as expected from this primarily auditory task, the visual regions are not in the top 15% of the regions with the highest nonreversibility.

Similarly, the WM task shows high levels of driving in regions including the MPFC, posterior cingulate, and the occipital-parietal junction, fully consistent with the literature (Barch et al., 2013; Drobyshevsky et al., 2006). In particular recent studies have demonstrated the importance of activity in MPFC for memory guided decision-making (Murphy et al., 2018, 2019). In these tasks, the decision is not possible based on the received input and thus demonstrates how the DMN orchestrates behaviour. In addition, the activity in the MPFC was also linked to self-reports indicating a greater focus on task-relevant detail (Murphy et al., 2019; Turnbull et al., 2019).

The findings support the view that the spatiotemporal dynamics of the brain cannot be adequately captured by solely separable intrinsic and task-evoked dichotomy, but rather a dynamic interplay of task-appropriate functional reconfigurations (Bolt et al., 2018).

### Heterogeneity

We further investigated the heterogeneity found at the node level by comparing the TENET results to various known forms of heterogeneity such as the gene expressions maps obtained from the Allen Human Brain Atlas (Arnatkeviciute et al., 2019; Deco et al., 2021a; Fornito et al., 2019; Hawrylycz et al., 2012). We found a significant correlation between the PCA genes values and node levels in the SOCIAL task, but not with the node levels found in REST (see Figure S1). The reverse was true for excitation-inhibition ratio given by the gene expression, which was correlated with the node levels in REST but not in SOCIAL. These significant differences between rest and task show how the environment is directing changes in the functional hierarchy. It is of considerable interest that the E-I ratio is significantly correlated with node levels in REST and not the node levels in task, since this suggest that REST is more intrinsically shaped. On the other hand, the significant correlation between the node levels in the SOCIAL task (but not with REST), suggests that the level of driving by the environment is not fully free but still constrained to a certain degree by genetics (here captured by the first PCA of the genes).

Further investigating the heterogeneity, we compared REST and SOCIAL directly to the myelinisation ratio (T1w/T2w ratio), which contains high values in the sensory regions of visual, somatomotor, and auditory (Burt et al., 2018). We did not find a correlation to the node levels of nonreversibility for both REST and SOCIAL task. This result clearly demonstrates that the environment is not driving the brain to nonreversibility by solely affecting the sensory regions but equally through driving higher associative brain regions across the whole brain. This fits well with the evidence that the three sources of heterogeneity investigated here are correlated among themselves (Burt et al., 2018; Deco et al., 2021a), yet influencing the functional hierarchy at the node level of nonreversibility differently under different conditions. Overall, these results provide evidence for the severe constraints in terms of degrees of freedom that the brain has to operate within.

### Node-Level Investigation of Neuropsychiatric Disorders

As shown above, at the global level, the three neuropsychiatric patient groups (ADHD, schizophrenia, and bipolar disorder) had lower levels of nonreversibility compared to healthy participants. Further investigating this finding, we also found significant local heterogenous node-level changes differentiating between the different disorders. Interestingly, in schizophrenia compared to controls, we found local decreases across the brain but primarily located in the temporal, parietal, and prefrontal cortices. These regions clearly have less nonreversibility compared to controls, which is compatible with the literature showing that the disorder is associated with more isolation, as a function of the loss of balance between intrinsic and extrinsic activity (van den Heuvel & Fornito, 2014).

In contrast, while in bipolar disorder the overall level of nonreversibility is lower than controls, the somatomotor regions show increases in nonreversibility, perhaps linked to the findings in the literature of large, sudden swings in mood, given that the brain is in more nonequilibrium (Furman et al., 2011; Menon & Uddin, 2010). Comparing ADHD with controls shows larger local levels of nonreversibility in somatosensory, temporal, parietal, and insular cortices. In particular, the somatomotor regions are more nonreversible than in controls, suggesting a potential route for hyperactivity, while the lower nonreversibility in parietal regions could be linked to the known attentional deficits in the disorder (Konrad & Eickhoff, 2010).

### Discussion of TENET Framework

The results produced by the TENET framework relies on two essential elements, namely, the concept of reversibility (as captured by the arrow of time) and how machine learning (and for example deep learning) is able to quantify the reversibility of brain signals. In terms of the arrow of time, this popularisation of this idea is usually credited to the physicist Arthur Eddington (Eddington, 1928). Here we showed that this key idea from physics and thermodynamics can equally well be applied in neuroscience. The second law of thermodynamics, as immortalised by Rudolph Clausius (Clausius, 1865) and Sadi Carnot (Carnot, 1824) states a nonequilibrium is characterised by the arrow of time which indicates the nonreversibility of a system. In fact, the second law of thermodynamics can be expressed by the Clausius inequality, which establishes that the work associated with the process (averaged over many repetitions) is larger than the change in its free energy, which is the same as stating that the system is nonreversible and in nonequilibrium.

As shown by Seif and colleagues, rather than computing the production entropy, the arrow of time can be cast as a game in which a player is shown either a forward or a backward trajectory and has to guess the direction of the arrow of time. The only information available is the trajectory, and from this a player must guess was generated by a forward or reverse process. The accuracy is then given by the ratio of correct predictions to the total number of samples. This statistical problem can be very hard when the fluctuations are high, and therefore the direction of the arrow of time is very difficult to ascertain (Crooks, 1998; Feng & Crooks, 2008; Jarzynski, 2011; Maragakis et al., 2008; Seif et al., 2021; Shirts et al., 2003). One way of solving this is to use a machine-learning algorithm trained such as deep learning to infer the direction of the arrow time. In fact, Seif and colleagues have demonstrated that classification between forward and reverse time series can identify the arrow of time in systems where the direction is known, and therefore establishes a one-to-one relationship between this classification, production entropy, and nonreversibility (Seif et al., 2021). Even more, Parrondo and colleagues demonstrated this relationship analytically (Parrondo et al., 2009).

However, given that the learning of the classifier could fail, this classification method is a necessary but not sufficient condition for assessing the arrow of time. Yet, as we show here this can be mitigated by using the same deep-learning network with the same training and generalisation data samples that balance the method and provide robust results when used to compare nonreversibility across different brain states. Note that other machine-learning methods (like SVM) could equally well be used. Indeed, as shown by de la Fuente and colleagues, different machine-learning architectures can successfully be used to determine the temporal irreversibility of neural dynamics of different brain states (de la Fuente et al., 2023). Yet, not all machine-learning architectures are equally good. Hence, why we took great care to build a suitable deep-learning network utilising the same parameters for comparisons. We note that the main results obtained here are in full agreement with the published literature. Lynn and colleagues used a very different approach to measure the production entropy and arrived at the same result for a subset of the HCP data used here, namely, that task is more nonreversible than rest. A similar confirmation of this result (using the full HCP dataset) was also obtained using the INSIDEOUT framework of classifying forward and reverse time series of the same dataset but not using machine learning (Deco et al., 2022).

In terms of the deep-learning framework, this method has received a lot of attention over the last couple of years. This powerful machine-learning technique has proven highly useful for providing solutions to a number of difficult computational problems ranging from vision to playing Go (Sejnowski, 2018, 2020; Silver et al., 2018; Yang & Wang, 2020). However, some criticisms have been raised over the largely black box nature of these advances, which have had considerable practical utility for solving complex problems, but have produced little in way of new insight into *how* this is achieved mechanistically (Marcus, 2018). Recent research has, however, started to harness the power of deep learning for discovering useful underlying mechanisms (Seif et al., 2021).

Here, we were not aiming to use deep learning as a technique for revealing underlying brain mechanisms but rather simply as a tool for providing the level of the reversibility of the arrow of time in brain signals. In other words, in our framework the important question is to determine the level of distinction between forward and time-reversed back time series, but not *how* this is achieved. As such, here the black box nature of deep learning was not relevant for solving the nontrivial problem of determining nonreversibility.

As mentioned above, the TENET framework can be studied at all spatial scales. Here, however, we focused on three levels: global, system level, and node level, where the global level considers all the whole-brain signals, while the node level considers the signals in each brain region separately and the system level considers the typical large-scale resting-state networks (Biswal et al., 1995, 2010; Smith et al., 2013). This allowed us to focus on different aspects of nonequilibrium, that is, the interactions between the environment and different brain scales, ranging from whole-brain to large-scale networks and to regions. Indeed, the node level turned out to be a highly sensitive measure of quantifying and interpreting different cognitive brain states and differences between health and multiple diseases.

### Use of Thermodynamics for Assessing the Environment Through Brain Signals

The TENET framework is especially powerful, given that thermodynamics can quantify the influence of the environment by the level of nonreversibility. More broadly, this is related to a fundamental question in general biology, namely, how survival is a key characteristic of life and requires the ability to find order in a complex, largely disordered environment. As proposed by the Austrian physicist and Nobel Laurate Ernst Schrödinger, survival is predicated on avoiding equilibrium: “How does the living organism avoid decay? … By eating, drinking, breathing and … assimilating. The technical term is metabolism” (Schrödinger, 1944). The avoidance of decay thus requires nonequilibrium interactions with the complex environment—and the brain is at the heart of these interactions.

There is a long history of understanding how the brain is able to interact with the environment. The initial extrinsic perspective proposed that the brain is primarily, reflexively driven by momentary stimulation from the environment in a *task-driven* manner (Raichle, 2010; Sherrington, 1906; Yuste et al., 2005). A more recent, complementary perspective was proposed by Marcus Raichle, which holds that the brain is mainly intrinsic, *resting* but switching between states whilst “interpreting, responding to, and even predicting environmental demands” (Raichle, 2006; Raichle et al., 2001). The evidence is clear that the brain’s metabolic energy budget for maintaining the intrinsic resting activity is large (Zhang & Raichle, 2010). In fact, by some estimates, 20% of the total energy consumption is taken up by the brain, which only represents 2% of body weight (Attwell & Laughlin, 2001; Clarke & Sokoloff, 1999; Magistretti et al., 1999), which has led to Raichle’s poetic proposal of “dark energy” (Raichle, 2006).

The brain’s energy budget governs the flow of energy between the brain and environment, which is the ultimate cause of the nonequilibrium essential to the proposals of Schrödinger and Raichle. Any living system requires the breaking of the detailed balance of the transitions between the underlying states (Gnesotto et al., 2018; Schrödinger, 1944). In a system with detailed balance, the fluxes of transitions between different states disappear (Lynn et al., 2021; Sanz Perl et al., 2021). This is conveniently described in the language of thermodynamics, where a system ceases to produce entropy and becomes reversible in time (Jarzynski, 2011). In contrast, a nonequilibrium system—where the balance is broken—shows net fluxes between the underlying states, and thus becomes irreversible, establishing an arrow of time (Crooks, 1998; Feng & Crooks, 2008; Maragakis et al., 2008; Seif et al., 2021; Shirts et al., 2003). This is closely linked to turbulence, a classical example of nonequilibrium, which has been shown to be highly useful for optimally transferring energy/information over space-time due to its mixing properties (Frisch, 1995). Turbulence has recently been demonstrated in the human brain, where the resulting information cascade is crucial for extracting order from disorder (Deco & Kringelbach, 2020; Sheremet et al., 2019).

The ideas for the present framework comes from physics and thermodynamics, where nonequilibrium is intrinsically linked to nonreversibility (Seif et al., 2021) and the production of entropy, leading to the arrow of time, as originally popularised by Arthur Eddington (Eddington, 1928) and since studied in great detail (Crooks, 1998; Feng & Crooks, 2008; Jarzynski, 2011; Maragakis et al., 2008; Seif et al., 2021; Shirts et al., 2003). In fact, a simple yet powerful way of assessing nonequilibrium in the brain is to quantitatively estimate the arrow of time of the brain signals rather than the more difficult way of estimating the production of entropy (Lynn et al., 2021; Sanz Perl et al., 2021).

The nonreversibility of a physical process and the arrow of time is clearly illustrated when watching a film of a glass being shattered, which is very different from watching the same film in reverse. In thermodynamics, this can be elegantly described in terms of the entropy production, which increases when a system becomes disordered. If the total entropy production is larger than zero, it means that the system is nonreversible and in nonequilibrium. In cases such when a glass is being shattered, the nonreversibility is very clear for all to see. In contrast, a film of colliding billiards balls can be watched equally forward and backward, making it very difficult to distinguish the correct arrow of time in the film. This process could potentially be fully reversible and not producing entropy.

In most processes, however, like the evolution of brain signals, the level of reversibility is much less clear. Here, we therefore used the power of deep machine learning to detect the level of reversibility of empirical brain-imaging data. This allowed us to assess the level of nonequilibrium in brain dynamics in different states. Specifically, for whole-brain data we extracted the normal forward time series as well as constructing the time reversal of the backward time series for a given parcellation. This procedure provides a clear arrow of time for a given time series for which a deep-learning classifier (here named Temporal Evolution NETwork, TENET) could be trained to identify the forward and the time reversal for a given time series of any length. The performance of TENET provides a reliable measure of the degree of nonreversibility and nonequilibrium at different levels of global and local brain organisation.

### Perspectives

The present thermodynamics-based deep-learning TENET framework opens up for an examination of differences of nonreversibility for different levels of consciousness, from sleep and anaesthesia to altered states of consciousness induced by psychedelics and meditation. The framework can easily be extended to other neuroimaging modalities such as MEG/EEG. It can even be used with LFP and other types of cell recordings in animals.

One particularly challenging question relates to the role of brain processing of reversible external stimuli such as, for example, watching the forward and backward versions of the movie of a glass being shattered. How will the nonreversibility in brain dynamics change with identical stimuli, but where the order has been changed such that the arrow of time has been violated? Will this elicit different nonreversibility in brain dynamics when showing the forward and backward versions of a movie of billiard balls moving, which is not in any clear way violating the arrow of time? These stimuli are experienced radically different, which must be linked to the interactions between the extrinsic stimulation with the dynamics of intrinsic predictability formed by prior experiences. However, intrinsic predictability is known to be affected in neuropsychiatric disease, and it would be of considerable interest to study how the reversibility of external stimulation influences the nonreversibility in brain dynamics. In fact, this could potentially reveal new information about the interactions between intrinsic and extrinsic dynamics, which are known to be compromised. The reversal of external stimuli could also take place at higher cognitive levels, such as when inverting the nodes of Bach’s fugue or that of a complex narrative.

From its conception, the arrow of time is coupled to the deep notion of causality. Thermodynamics offers important tools for establishing the causal directionality of information flow through the concept reversibility and entropy. There is of course a large literature on causality, best summarised in the seminal work by Judea Pearl (Pearl, 2009), where he shows that any framework of causal inference is based on inferring causal structures that are equivalent in terms of the probability distributions they generate; that is, they are indistinguishable from observational data, and could only be distinguished by manipulating the whole system.

In neuroscience, there have been numerous attempts to capture causality in brain dynamics. One influential concept is ignition, the idea that a stimulus can ignite a causal chain of events propagating across the brain (Dehaene et al., 1998). This ignition can happen as a result of extrinsic stimulation (Joglekar et al., 2018; Mashour et al., 2020; van Vugt et al., 2018) or as part of intrinsic events (Deco et al., 2017, 2021a). More sophisticated approaches use probabilistic principles of mutual information (Brovelli et al., 2015; Pereda et al., 2005; Quiroga et al., 2000, 2002) to determine the directional causality underlying the functional hierarchical organisation of brain function (Deco et al., 2021b).

The concept of the arrow of time has also been investigated from the perspective of chaos theory, originating with the work of Henri Poincaré who published the first description of chaotic motion in 1890 (Poincaré, 1890). Later work has confirmed that one key characteristic of chaos is the infinite sensitivity to initial conditions (Strogatz, 2018). Given this extreme sensitivity, even if a classic mechanic deterministic chaotic system is in principle reversible, in practice this is in fact nonreversible. In other words, chaos makes it very difficult to establish the computational reversibility and thus causality. Turbulence is a classic example of a spatiotemporal chaotic system that is associated with nonequilibrium and thus nonreversibility. Interestingly, turbulence is a highly useful dynamical regime for optimally transferring energy/information over space-time, and it has recently been shown that brain dynamics are indeed turbulent (Deco & Kringelbach, 2020). The turbulent regime supports the information cascade that is crucial for extracting order from disorder.

Overall, the novel thermodynamics-based deep-learning TENET framework can provide detailed information of the varying levels of nonstationary and nonequilibrium nature of brain dynamics in health and disease. The TENET framework offers a quantitative account of differences in nonreversibility. Future work could integrate this with causal mechanistic whole-brain modelling in a turbulent regime to deepen our understanding of how brain dynamics organise human behaviour in the face of the second law of thermodynamics in health and disease.

## METHODS

### Neuroimaging Ethics

For the HCP dataset, the Washington University–University of Minnesota (WU-Minn HCP) Consortium obtained full informed consent from all participants, and research procedures and ethical guidelines were followed in accordance with Washington University institutional review board approval.

For the UCLA dataset, as detailed in Poldrack et al. (2016), the Consortium for Neuropsychiatric Phenomics recruited neuropsychiatric participants and healthy controls who gave written informed consent following procedures approved by the Institutional Review Boards at UCLA and the Los Angeles County Department of Mental Health.

### Neuroimaging Participants HCP Rest and Task

The dataset used for this investigation was selected from the March 2017 public data release from the HCP where we chose a sample of 990 participants from the total of 1,003 participants, since not all participants performed all tasks.

### The HCP Task Battery of Seven Tasks

The HCP task battery consists of seven tasks: working memory, motor, gambling, language, social, emotional, and relational, which are described in details on the HCP website (Barch et al., 2013). HCP states that the tasks were designed to cover a broad range of human cognitive abilities in seven major domains that sample the diversity of neural systems: (1) visual, motion, somatosensory, and motor systems; (2) working memory, decision-making, and cognitive control systems; (3) category-specific representations; (4) language processing; (5) relational processing; (6) social cognition; and (7) emotion processing. In addition to resting-state scans, all 1,003 HCP participants performed all tasks in two separate sessions (first session: working memory, gambling and motor; second session: language, social cognition, relational processing, and emotion processing). As a test-retest control condition, a small subsample of 45 HCP participants performed the paradigm twice.

### Neuroimaging Participants UCLA Rest

Consortium for Neuropsychiatric Phenomics published a dataset with neuroimaging as well as phenotypic information for 272 participants. We used the preprocessed data with a total of 261 participants, since seven of the participants were missing T1-weighted scans (Gorgolewski et al., 2017), and three healthy controls and one ADHD patient were missing resting-state scans. The total population analysed consists of 122 healthy controls, as well as participants with diagnoses of adult ADHD (40 patients), bipolar disorder (49 patients), and schizophrenia (50 patients).

### Neuroimaging Structural Connectivity and Extraction of Functional Time Series

#### HCP preprocessing and extraction of functional time series in fMRI resting-state and task data.

The preprocessing of the HCP resting-state and task datasets is described in detail on the HCP website. Briefly, the data is preprocessed using the HCP pipeline, which is using standardized methods using FSL (FMRIB Software Library), FreeSurfer, and the Connectome Workbench software (Glasser et al., 2013; Smith et al., 2013). This standard preprocessing included correction for spatial and gradient distortions and head motion, intensity normalization and bias field removal, registration to the T1 weighted structural image, transformation to the 2-mm Montreal Neurological Institute (MNI) space and using the FIX artefact removal procedure (Navarro Schröder et al., 2015; Smith et al., 2013). The head motion parameters were regressed out, and structured artefacts were removed by ICA + FIX processing (independent component analysis followed by FMRIB’s ICA-based X-noiseifier (Griffanti et al., 2014; Salimi-Khorshidi et al., 2014). Preprocessed time series of all grayordinates are in HCP CIFTI grayordinates standard space and available in the surface-based CIFTI file for each participants for resting-state and each of the seven tasks.

We used a custom-made MATLAB script using the ft_read_cifti function (Fieldtrip toolbox; Oostenveld et al., 2011) to extract the average time series of all the grayordinates in each region of the DK80 parcellation, which are defined in the HCP CIFTI grayordinates standard space. The BOLD time series were filtered using a second-order Butterworth filter in the range of 0.008–0.08 Hz.

#### UCLA preprocessing and extraction of functional time series in fMRI resting-state data.

The preprocessing of UCLA resting-state datasets is described in detail on the website and in the paper by Gorgolewski and colleagues (Gorgolewski et al., 2017). Briefly, the preprocessing was performed using FMRIPREP version 0.4.4 (https://fmriprep.readthedocs.io). This robust preprocessing pipeline is based on the Nipype workflow engine7 and aims to combine different implementations of various MR signal processing algorithms (from established software packages) to deliver a robust spatial normalization and nuisance estimation workflow.

### Parcellations

For the analysis at the global and node level, all neuroimaging data was processed using the DK80 standard parcellations (Deco et al., 2021b). Briefly, this was constructed using the Mindboggle-modified Desikan–Killiany parcellation (Desikan et al., 2006) with a total of 62 cortical regions (31 regions per hemisphere) (Klein & Tourville, 2012). We added the 18 subcortical regions, that is, nine regions per hemisphere: hippocampus, amygdala, subthalamic nucleus (STN), globus pallidus internal segment (GPi), globus pallidus external segment (GPe), putamen, caudate, nucleus accumbens, and thalamus. This provided a total of 80 regions in the DK80 parcellation, also precisely defined in the common HCP CIFTI grayordinates standard space. For the analysis at the system level we also used the Schaefer500, where each parcel is marked with the seven resting-state network in the Yeo7 parcellation (Schaefer et al., 2018; Yeo et al., 2011).

### TENET Deep-Learning Framework and Associated Methods

The TENET deep-learning framework is a general method that can use many types of data. However, it is important that the data for training and generalisation are balanced between different conditions. We used TENET to carry out two independent analyses using two different datasets from HCP and UCLA, and below we show how we avoided any potential problems with bias.

### HCP Dataset

For the large-scale HCP dataset, we performed the reversibility and nonequilibrium analyses at three different spatial scales: *Global*, *system level*, and *local node level*. For each scale, we trained and analysed three different TENETs, one for each spatial scale. We used roughly 90% of the data for the training set (890 participants) and the remaining 10% for the test and validation sets (100 participants). Note that the sliding windows used for training belong to different participants than the ones used for test and validation, hence there are no overlapping windows.

For the global level, the input patterns consisted of the windowed time series across the whole brain in the DK80 parcellation, that is, each pattern consisted of 80 windowed time series.

For the system level, the input patterns consisted of the windowed time series in the Schaefer500 parcellation, that is, each parcel belonged to one of the seven Yeo resting-state networks. We performed the analysis for the Schaefer500 parcels belonging to each of the seven Yeo networks and thus obtained a measure of nonequilibrium for each Yeo network.

Finally, for the finest spatial scale at the node level, the input patterns consisted of the windowed time series for one parcel in the DK80 parcellation, that is, each of the patterns consisted of one windowed time series for that parcel. We performed this for each of the 80 parcels in the DK80 separately such that we obtained a measure of nonreversibility for each brain region. For the analysis of the HCP data, we carried out these three spatial-scale analyses for HCP rest and seven tasks, that is, a total of 24 different analyses.

The input to TENET consisted of sliding windows from the BOLD neuroimaging data from all the participants, shortened to the shortest duration of a task (EMOTION task with 176 TRs), always starting from the beginning. We generated these sliding windows with slightly different parameters for the three types of analysis: for global and system-level analysis, we used a window size of 20 TRs (each lasting 0.72 sec), and for node-level analysis, we used a window size of 150 TRs. These were then shifted forward until the end of the datasets (with a length of 3 TRs for global and system-level analysis and 2 TRs for node-level analysis). In all cases, the parameters (window size and overlap) were found by varying them and finding the most robust results for generalisation.

As an example of how we chose the two parameters for each level of analysis, Supporting Information Table S1 shows the global-level analysis for all resting-state data in all participants, the Accuracy and Information Loss for the Training set, and the Accuracy and Information Loss for the Cross-validation Set using varying window sizes [10, 20, 30] and overlaps [1, 3, 6] (both in TRs). In general, as can be seen, the best results were found for window sizes [10, 20], TRs, and overlaps [3, 6]. Our criteria were to take the smallest overlap and the largest possible window size, hence why we chose a window size of 20 TRs with an overlap of 3 TRs. Nevertheless, the results are stable and robust within this range.

For each of the sliding windows, we generated two input patterns containing (1) forward and (2) time-reversed backward-sliding windowed time series, and for the supervised learning, we associated each pattern with the output class label of forward and backward, respectively.

### UCLA Dataset

For the analysis of the smaller scale UCLA dataset with coarser TR (2 sec) and shorter duration (152 TRs), we carried out different spatial-scale analyses at the level of global and node level for the resting-state data of control and three neuropsychiatric disorders (schizophrenia, bipolar disorder, and ADHD). For this much smaller dataset of 261 participants, we used roughly 90% of the data for the training set (40 participants in each of the four conditions) and the remaining data for the test and validation sets (i.e., 36 participants for the global and 35 participants for node-level analysis). For the global analysis, we shuffled the data 500 times in order to perform nonparametric significance testing (for the box plot in Figure 6A). For the node analysis we shuffled the data 10 times and computed the average node level of nonreversibility. We do not provide statistics for the node analysis, and note that it is computationally very expensive to add more shuffles. Nevertheless, we show the rendered node-level results on a qualitative basis.

The input to TENET consisted of sliding windows from the BOLD neuroimaging data from all the participants. We generated these sliding windows with slightly different parameters for the two types of analysis: for global-level analysis, we used a windows size of 10 TRs of 2 sec and for node-level analysis, we used a window size of 20 TRs. These were then shifted forward (in steps of 1 TR) until the end of the dataset.

### Training and Generalisation in Deep-Learning Network TENET

This data was then used with TENET for training and generalisation in the following way. After training, we measure the reversibility of each pattern in the validation set. This is computed by comparing the trained output for the forward and backward versions of each pattern. More specifically, for all three spatial scales, we compute the level of reversibility, *R*(*t*), for a given sliding window at time *t* by using the following equation:

$$R\left(t\right)=\frac{\left(o_{\text{forward}}\left(2\right)-o_{\text{forward}}\left(1\right)\right)+\left(o_{\text{backward}}\left(1\right)-o_{\text{backward}}\left(2\right)\right)}{2}$$

Here *o*_*forward*_(*i*) is the output *i* of the final output classification layer, when the forward pattern is presented in the input layer. Similarly, *o*_*backward*_(*i*) is the output for node *i* of the final output classification layer, but now when a backward pattern is presented in the input layer. Given that the forward and backward categories are associated with the outputs *o*_*forward*_ = [0, 1] and *o*_*backward*_ = [1, 0], respectively, *R*(*t*) thus represents the degree of reversibility for a particular sliding window at time *t*. If either the degrees of right classification of forward (*o*_*forward*_(2) − *o*_*forward*_(1)) or backward (*o*_*backward*_(1) − *o*_*backward*_(2)) are smaller than 0, this means that the classification is incorrect and in this case we set *R*(*t*) = 0. The degree of reversibility *R*(*t*) is measuring the degree of nonreversibility at time *t*. We also report the mean and the standard deviation over time, which corresponds to what is called the mean nonreversibility and the standard deviation of nonreversibility (across time) in the results and figures.

For the architecture of TENET, we use the standard MATLAB architecture with an input layer of size *N* * *w*, where *N* corresponds to the spatial scale (i.e., global *N* = 1, system level *N* = 7, node level *N* = 80), and *w* is the size of the sliding window. This is followed by 10 fully connected layers including (1) batch normalisation, which normalises a minibatch of data across all observations for each channel independently, and (2) a nonlinearity operation implemented using *reluLayer*, which performs a threshold operation to each element of the input, where any value less than zero is set to zero. The dimensions of the 10 layers are [2048; 1024; 512; 256; 128; 64; 32; 16; 8; 4]. The final layer of TENET is a softmax classification layer of dimension [2], corresponding the two possible output class labels (forward and backward).

For training TENET, we used the deep-learning algorithm ADAM, which is an algorithm for first-order gradient-based optimization of stochastic objective functions, based on adaptive estimates of lower order moments to attenuate the effects of noise as it is implemented in MATLAB with the recommended default parameters (Kingma & Ba, 2014). These standard MATLAB training options are as follows: GradientDecayFactor: 0.9000, SquaredGradientDecayFactor: 0.9990, Epsilon: 1.0000e-08, InitialLearnRate: 1.0000e-03, LearnRateSchedule: ‘none’, LearnRateDropFactor: 0.1000, LearnRateDropPeriod: 10, L2Regularization: 1.0000e-04, GradientThresholdMethod: ‘l2norm’, GradientThreshold: Inf, MaxEpochs: 10, MiniBatchSize: 128, Verbose: 1, VerboseFrequency: 50, ValidationData: [],ValidationFrequency: 30, ValidationPatience: Inf. Shuffle: ‘once’, CheckpointPath: “, ExecutionEnvironment: ‘auto’, WorkerLoad: [], OutputFcn: [],Plots: ‘none’, SequenceLength: ‘longest’, SequencePaddingValue: 0, SequencePaddingDirection: ‘right’, DispatchInBackground: 0, ResetInputNormalization: 1.

### Measuring the Breaking of Detailed Balance of the System

We wanted to test if nonreversibility is associated with the breaking of detailed balance of a system. In order to show the increase in nonbalance in nonequilibrium, we decided to characterise the level of asymmetry in the interaction as expressed by the shifted correlation matrix. Specifically, we selected patterns from sliding windows of low and high reversibility, and computed the time-delayed functional connectivity matrix between all pairs of brain regions, *FC*(*τ*)

$${FC}_{ij}\left(\tau \right)=<x_{i}\left(t\right),x_{j}\left(t+\tau \right)>$$

where <> indicates correlation over time of the fMRI time series *x* and we used *τ* = 3 (in TRs), which was selected using the autocorrelation function, such that the chosen delay produced sufficient decay. We computed the *FC*_*ij*_(*τ*) over all participants and all sliding windows for each condition (for HCP REST and the SOCIAL task, the most nonreversibility task). For the global level of asymmetry, we computed for each sliding window the degree of asymmetry as the mean value of the elements of the matrix: *FC*_*ij*_(*τ*) − *FC*_*ij*_(*τ*)*^T^*, where the superscript *T* indicates the transposition. For the node level of asymmetry, we first computed for each sliding window, the incoming flow *F*_*in*_(*i*) = ∑_*j*_*FC*_*ij*_(*τ*), and the outcoming flow *F*_*out*_(*j*) = ∑_*i*_*FC*_*ij*_(*τ*). The node level of asymmetry for node *i* is then given by <|*F*_*out*_(*i*) − *F*_*in*_(*i*)|>_*t*_, where <>_*t*_ indicates the average of the node-level asymmetry over all sliding windows and participants. We render the change between high and low levels of the node-level asymmetry.

### Support Vector Machine for System-Level Classification

We used a SVM with Gaussian kernels as implemented in the MATLAB function *fitcecoc*. The function returns a full, trained, multiclass, error-correcting output codes (ECOC) model. This is achieved using the predictors in the input with class labels. The function uses *K*(*K* − 1)/2 binary SVM models using the one-versus-one coding design, where we used *K* = 8 as the number of unique class labels. In other words, the SVM had seven inputs (the Yeo resting-state networks) corresponding to the output produced by the system-level TENET. The output was eight classes corresponding to the conditions (rest and seven tasks). We used the output from the 100 HCP participants used for generalisation, subdivided into 90% training and 10% validation, repeated and shuffled 100 times.

## ACKNOWLEDGMENTS

G. D. is supported Spanish national research project (ref. PID2019-105772GB-I00 MCIU AEI) funded by the Spanish Ministry of Science, Innovation and Universities (MCIU), State Research Agency (AEI); HBP SGA3 Human Brain Project Specific Grant Agreement 3 (grant agreement no. 945539), funded by the EU H2020 FET Flagship programme; SGR Research Support Group support (ref. 2017 SGR 1545), funded by the Catalan Agency for Management of University and Research Grants (AGAUR); Neurotwin Digital twins for model-driven noninvasive electrical brain stimulation (grant agreement ID: 101017716) funded by the EU H2020 FET Proactive programme; euSNN European School of Network Neuroscience (grant agreement ID: 860563) funded by the EU H2020 MSCA-ITN Innovative Training Networks; CECH The Emerging Human Brain Cluster (Id. 001-P-001682) within the framework of the European Research Development Fund Operational Program of Catalonia 2014–2020; Brain-Connects: Brain Connectivity during Stroke Recovery and Rehabilitation (id. 201725.33) funded by the Fundacio La Marato TV3; Corticity, FLAG–ERA JTC 2017 (ref. PCI2018-092891) funded by the Spanish Ministry of Science, Innovation and Universities (MCIU), State Research Agency (AEI). MLK is supported by the Center for Music in the Brain, funded by the Danish National Research Foundation (DNRF117), and Centre for Eudaimonia and Human Flourishing at Linacre College funded by the Pettit and Carlsberg Foundations.

## SUPPORTING INFORMATION

Supporting information for this article is available at https://doi.org/10.1162/netn_a_00300. The multimodal neuroimaging data are freely available from HCP. The UCLA data are freely available from the Consortium for Neuropsychiatric Phenomics. The code used to run the analysis is available on GitHub (https://github.com/decolab/tenet).

## AUTHOR CONTRIBUTIONS

Gustavo Deco: Conceptualization; Data curation; Formal analysis; Funding acquisition; Investigation; Methodology; Project administration; Resources; Software; Supervision; Validation; Visualization; Writing – original draft; Writing – review & editing. Yonathan Sanz Perl: Methodology; Software; Writing – original draft; Writing – review & editing. Laura de la Fuente: Methodology. Jacobo D. Sitt: Methodology. Thomas Yeo: Methodology; Writing – review & editing. Enzo Tagliazucchi: Conceptualization; Methodology; Writing – review & editing. Morten Kringelbach: Conceptualization; Data curation; Formal analysis; Funding acquisition; Investigation; Methodology; Project administration; Resources; Software; Supervision; Validation; Visualization; Writing – original draft; Writing – review & editing.

## FUNDING INFORMATION

Morten Kringelbach, Danmarks Grundforskningsfond (https://dx.doi.org/10.13039/501100001732), Award ID: DNRF117.

## TECHNICAL TERMS

Hierarchy: Here defined as the asymmetrical relationship between forward and backward interactions between brain regions.
Thermodynamics: A branch of physics dealing with the relationship between heat and other forms of energy, such as work. Here used to measure the transfer of energy/information from one brain region to another, and the ways in which this transfer affects the brain.
Detailed balance: In thermodynamics, a concept describing the equilibrium state of a system in which the forward and reverse reactions are occurring at the same rate.
Production entropy: A measure of the uncertainty or unpredictability of a process. The amount of production entropy produced in any nonreversible processes directly measures the asymmetry in time of the evolution of states in a nonequilibrium system.
Nonequilibrium: A system out of equilibrium.
Nonreversibility (or irreversibility): The asymmetry in the flow of events, also known as the ‘arrow of time’.
Deep learning: A broad class of machine-learning methods based on artificial neural networks able to learn from data in a hierarchical manner using multiple layers of interconnected nodes to extract increasingly complex features of the data at each layer.
Clausius inequality: The mathematical relationship describing the relationship between heat and work in a thermodynamic system and stating that the change in entropy of a system is always greater than or equal to the amount of heat transferred to the system, divided by the temperature at which the heat transfer occurs.
Free energy: A thermodynamic concept, also known as Gibb’s free energy, used to describe the amount of energy in a system that is available to do work.
Machine learning: A subset of artificial intelligence involving the use of algorithms and statistical models to enable a system to improve its performance on a specific task over time.
