# Supplementary material for: The arrow of time of brain signals in cognition: Potential intriguing role of parts of the default mode network
Source: Netw Neurosci. 2023 Oct 1;7(3):966–98. doi: 10.1162/netn_a_00300 (PMC10473271; doi:10.1162/netn_a_00300)
Supplement: Supplementary file 1 [file netn-7-3-966-s001.pdf]

Deco, G., Perl, Y. S., de la Fuente, L., Sitt, J. D., Yeo, B. T. T., Tagliazucchi, E., Kringelbach, M. L. (2022). Supporting information for "The arrow of time of brain signals in cognition: potential intriguing role of parts of the default mode network." *Network Neuroscience*. Advance publication. https://doi.org/10.1162/netn_a_00300

Supplementary material

The arrow of time of brain signals in cognition: Potential intriguing role of parts of the default mode network

Gustavo Deco[1-4], Yonatan Sanz Perl[1,5], Laura de la Fuente[5], Jacobo D. Sitt[6], B.T. Thomas Yeo[7], Enzo Tagliazucchi[5,8] and Morten L. Kringelbach[9-11]

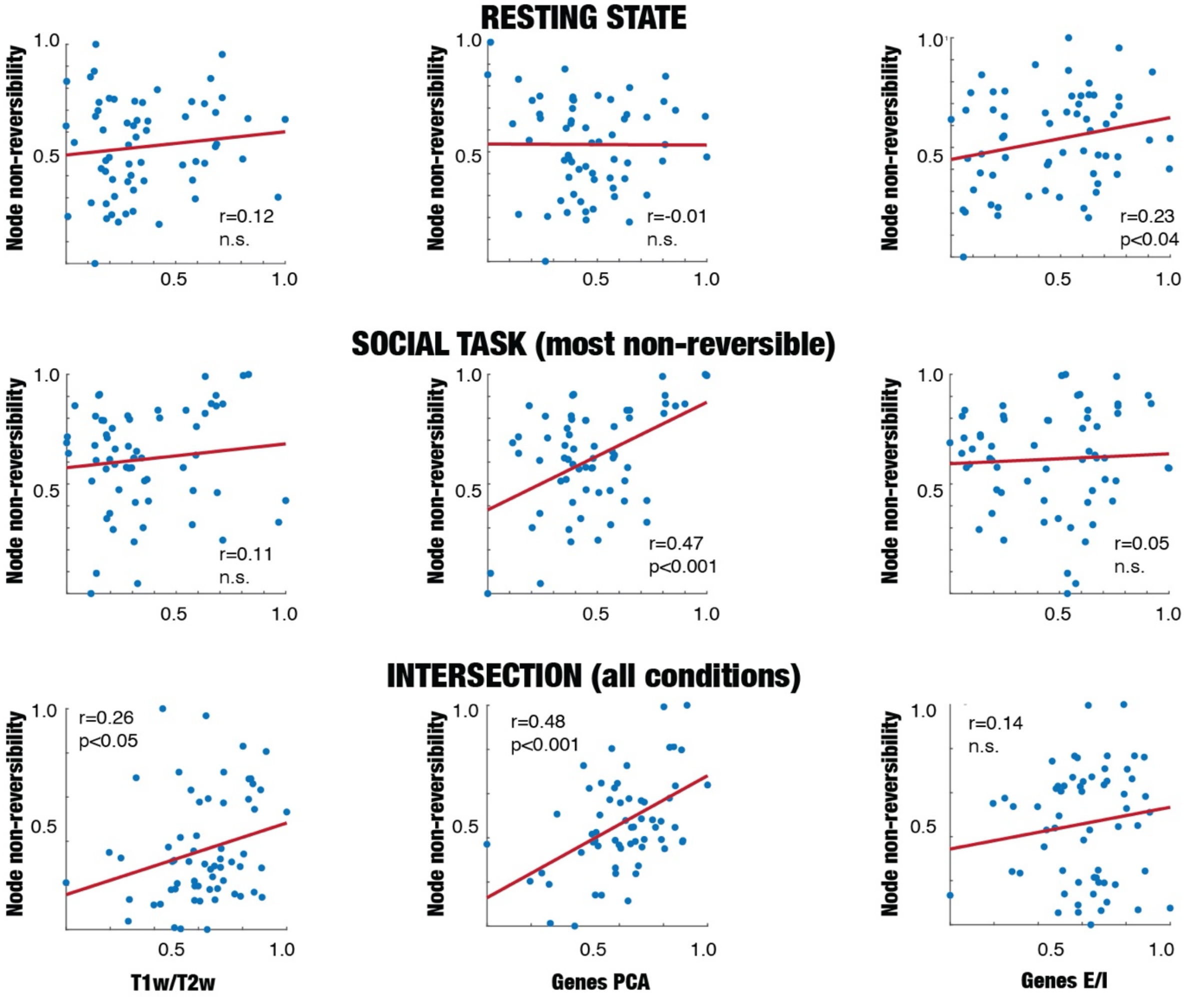

Figure S1. Hierarchy and heterogeneity at the node-level for resting state, most non-reversible task and intersection of all conditions. *We studied the hierarchy of the mean levels (across participants) of non-reversibility for rest, most non-reversible task and intersection by comparing this to other known sources of heterogeneity such as the myelinisation ratio (T1w/T2w ratio, obtained from HCP data) and various forms of gene expressions in the brain, obtained from the Allen Human Brain Atlas (Arnatkeviciute et al., 2019; Deco et al., 2021a; Fornito et al., 2019; Hawrylycz et al., 2012). In particular, we used the first PCA component of all genes and the excitation/inhibition ratio given by the gene-expression for genes coding for the excitatory AMPA and NMDA receptors and inhibitory GABA-A receptor isoforms and subunits. In the resting state condition, we found a significantly correlation (p<0.04, non-parametric) with the excitation-inhibition ratio but not with the two other measures. In contrast, the SOCIAL task showed a clear significant correlation with the first PCA (p<0.001, non-*

parametric) but not with the others. In contrast, we found significant correlations between the intersection of non-reversibility of all conditions with myelin ($p<0.05$, non-parametric) and Genes PCA ($p<0.001$, non-parametric). This bolsters the role of the DMN for orchestrating cognition.

Accuracy Training Set

Overlap \ Window sizes	10	20	30
1	89.3	92.2	90.6
3	94.6	95.2	95.5
6	96.2	95.3	92.6

Accuracy Cross-validation Set

Overlap \ Window sizes	10	20	30
1	73.4	73.2	69.5
3	78.5	77.4	74.7
6	80.3	79.9	73.4

Information Loss Training Set

Overlap \ Window sizes	10	20	30
1	0.25	0.19	0.22
3	0.13	0.12	0.12
6	0.10	0.12	0.20

Information Loss Cross-validation Set

Overlap \ Window sizes	10	20	30
1	0.65	0.73	0.77
3	0.63	0.65	0.73
6	0.58	0.55	0.68

***Table S1*. Example of selection of window size and overlap parameters for the global-level analysis in resting state.** The table shows the Accuracy and Information Loss for the Training set, and the Accuracy and Information Loss for the Cross-validation Set using varying window sizes [10,20,30] and overlaps [1,3,6] (both in TRs).
